# The Book World of Medicine and Science

**Published:** 1893-07-29

**Authors:** 


### THE BOOK WORLD OF MEDICINE AND SCIENCE.

### BOOK REVIEWS.

THE LAW OF CREMATION. An Outline of the Law relating to Cremation, Ancient and Modern, together with the rules and regulations of various Cremation Societies at Home and Abroad. By AUBREY RICHARDSON, Solicitor. (London: Reeves and Turner. 1893.)

This is an attempt to show what power an individual has to determine the manner of disposal of his own dead body. The writer is a lawyer, the son of Sir Benjamin Ward Richardson, and he has extended his inquiry far beyond the scope of the question he originally essayed to answer, and has collected a great amount of interesting and useful information on the subject of cremation. There is no department of the social organisation in which reforms or modifications are brought about with so much difficulty as in the disposal of the dead. Sentiment, the most unsafe of all guides to conduct, is in supreme authority when death enters a family, and the voice of reason is only too often hushed. It is this fact that chiefly explains the small progress that cremation has hitherto made among us. It is a violent change in our customs, runs counter to certain religious convictions most tenaciously held by some people, and although justified by strong scientific arguments, is entirely devoid of sentimental attractions. It requires a very strong conviction of its rightness to make anyone face the prejudice that at present rises against cremation. The advocates of cremation must not be daunted by the difficulties in their path; and we welcome this book of Mr. Richardson's as one which will increase the popular knowledge of the practice, and also be of use in clearing up the legal difficulties that may arise. As the question of cremation is one that medical men ought to be well acquainted with, and is a practice with which they must be in sympathy, we gladly bring this book to their notice.

WORDS ON EXISTING RELIGIONS. By the Hon. ALBERT S. G. CANNING. (W. H. Allen and Co.)

Parliament was electrified, not so very long ago, by a sudden storm of indignation roused in an aged politician after months of iron self-restraint, by the simple remark that his government was not worth attacking. Why this assumption of a non-militant attitude should have had such an irritating effect it is hard to say. But a certain species of tolerance has upon many minds a very similar effect. The time has gone by when it was necessary to plead with Maurice for "tolerance of intolerance." A little vigorous bigotry has become positively refreshing. It is the vague self-complacent generalisations about other people's faith and duty, the lofty patronage of the bland philosophic "mugwump," in a word, it is tolerance itself, for which in the present day tolerance is needed. Mr. Canning has a few words to say in this volume on Parseeism, Brahminism, Buddhism, Judaism, Christianity, Mohammedanism, and modern Freethought. He thinks they are all excellent things in their way. In fact, he is quite pleased to think how many people are still under their influence, and what an amount of information can be gained about them, all without knowing so much as another language beside our own. He believes the tendency of the age is for men to

THE BOOK WORLD OF MEDICINE AND SCIENCE.—(*continued*).

admire more and more the religion of each other, while growing more and more delightfully indifferent to their own. He has read a good many little books about these things, of which he has kindly furnished the reader with a list. His erudition extends even to Macaulay's essays and Lemprière's Dictionary. With extracts "of sorts" the pages are prettily garnished, and with high sounding references, but the reader who should attempt to verify a reference from the "Decline and Fall" for instance, without indication of page or volume, must possess either the omniscience of Macaulay's schoolboy or patience worthy of a better object. The work is in fact a specimen of book-making. In the endeavour to sustain a flight higher than that of all existing religions, it never descends to the level of imparting information about any one of them, and it is altogether lacking in qualities of style which might help to atone for other deficiencies.

FIRST AID TO THE INJURED. Five Ambulance Lectures by Dr. FRIEDRICH ESMARCH, Professor of Surgery at the University of Kiel. Translated from the German by H.R.H. Princess Christian. Fifth Edition. (London: Smith, Elder, and Co. 1893.)

Ambulance lectures are now quite an institution, and their scope and intention are thoroughly understood. They have led to the publication of many manuals on the subject. This is one of the briefest and best of them. Professor Esmarch is a surgeon of great distinction, and he has paid special attention to many of those subjects which find a place in ambulance lectures, particularly field dressings, and the transport of the wounded. He has been particularly fortunate in his Royal translator, for Her Royal Highness has shown the truest sympathy with such instruction as these lectures are designed to give. The German Edition, of which this is a translation, has not been brought up to date in the part devoted to the antiseptic treatment of wounds. The old carbolic spray is spoken of as being in routine use, while as a fact it is as extinct in most hospitals as the dodo. We cannot but regret that care was not taken to rewrite much of this section. A curious error in one of the illustrations (fig. 6) has escaped notice; it is no less than placing the thumb on the inner (ulnar) side of the hand! As the figure is to illustrate the mode of compressing the brachial artery, such an error is of importance. All the four figures to illustrate the compression of arteries are faulty and unworthy of such an excellent manual.

LEWIS'S DIET CHARTS.

MR. LEWIS, of Gower Street, has brought under our notice a series of diet charts for handing to patients after consultation, modified to suit individual requirements. There are thirteen charts for as many different conditions constipation, diarrhœa, diabetes, albuminuria, gout, rheumatism, fevers, phthises, &c. They are drawn up to show both what the patient may take and what he may not take, the articles of diet being properly grouped, and blanks left for additions. The charts will be found useful, both in saving time, in adding precision to directions, and also in suggesting points to the physician. Our knowledge of the influence of particular articles of diet in different pathological conditions is still so imperfect that exact unanimity of opinion is unattainable, but we believe these charts will meet with very general approval and be found really useful.

